# Deltamethrin-Mediated Toxicity and Cytomorphological Changes in the Midgut and Nervous System of the Mayfly *Callibaetis radiatus*

**DOI:** 10.1371/journal.pone.0152383

**Published:** 2016-03-31

**Authors:** Yeisson Gutiérrez, Helen P. Santos, José Eduardo Serrão, Eugênio E. Oliveira

**Affiliations:** 1 Departamento de Entomologia, Universidade Federal de Viçosa, Viçosa, MG 36570–000, Brasil; 2 Departamento de Biologia Geral, Universidade Federal de Viçosa, Viçosa, MG 36570–000, Brasil; Federal University of Rio de Janeiro, BRAZIL

## Abstract

Immature instars of mayflies are important constituents of the food web in aquatic ecosystems (especially in Neotropical regions) and they are among the most susceptible arthropods to pyrethroid insecticides. These insecticides have been recognized as important stressors of freshwater ecosystems, but their cellular effects in aquatic insects have been neglected. Here, we assessed the susceptibility to deltamethrin (a typical type II pyrethroid) as well as the deltamethrin-mediated cytomorphological changes in the central nervous system and midgut of the mayfly *Callibaetis radiatus*. While the deltamethrin LC_50_ for 24h of exposure was of 0.60 (0.46–0.78) μg of a.i/L, the survival of *C*. *radiatus* was significantly reduced in deltamethrin concentrations ≥ 0.25 μg a.i/L at 96h of exposure. Sub-lethal deltamethrin exposure severely affected the cytomorphology of *C*. *radiatus* midgut (e.g., muscle layer retraction, cytoplasm vacuolation, nucleus and striated border disorganization) and also induced slight cytomorphological changes in the brain (e.g., presence of pyknotic nuclei) and in the thoracic ganglia (e.g., vacuolation of neurons and presence of pyknotic nuclei) of these insects. However, DNA damage was absent in all of these organs, suggesting that the sublethal cellular stress induced by deltamethrin might disrupt physiological processes (e.g., metabolism or electrical signal transmission) rather than cause cell death (e.g., apoptosis) in *C*. *radiatus*. Thus, our findings indicated that deltamethrin actions at cellular levels represent a clear indication of sublethal effects on the *C*. *radiatus* survival abilities.

## Introduction

The extensive use of pyrethroid insecticides in the control of virtually all agriculturally and medically important arthropod pests has raised concerns about its environmental safety, including their potential for serious harm in aquatic invertebrates [1–5]. The entry of this insecticide into aquatic ecosystems can occur *via* direct applications in water surfaces [6,7] or as a result of agricultural use, including spray drift, runoff and drainage [8–10].

Compared to organochlorine and other long-lasting compounds, insecticides such as the type II pyrethroid deltamethrin are rapidly broken down in sunlight [11,12]. However, such rapid sunlight-induced deltamethrin degradation has not affected its occurrence in water and sediments of aquatic ecosystems [13–16], increasing the possibility of deltamethrin intoxication for benthic organisms [16].

The main pyrethroid effects on insects are related to the physiological impairment of the voltage-gated sodium channels that are responsible for the initiation and propagation of action potentials in excitable cells [17–19]. This insecticide prolongs the opening of sodium channels, resulting in membrane depolarization leading to conductance block in the nervous system [20,21]. Other auxiliary targets, especially voltage-gated calcium and chloride channels, have been implicated in the actions of a subset of pyrethroids [19,22,23]. Furthermore, pyrethroid has been suggested to disrupt ion transport processes at epithelial tissues on aquatic and terrestrial insects [24–26].

Although mayfly species have become important model organisms in insecticide ecotoxicology [3,27–31], the insecticide-mediated cytomorphological changes in their central nervous system (the proposed target of the pyrethroids) and midgut (tissue of deltamethrin secondary actions) have been neglected. To address these knowledge gaps, we assessed the acute and chronic toxicity for deltamethrin in nymphs of the mayfly *Callibaetis radiatus* (Ephemeroptera: Baetidae). These ephemeropterans are well distributed in lentic environments of Neotropical regions [32], but have not been tested for pesticide susceptibilities. Furthermore, considering the fact that *Callibaetis* spp. nymphs are an important part of the food web in aquatic ecosystems [33] and other mayflies (e.g. Baetidae) have been used as biological indicator of environmental degradation [34], the findings of this study provide new insights that will assist in biomonitoring the influence of pesticides on benthic macroinvertebrate assemblages.

## Material and Methods

All applicable international, national, and institutional guidelines for the care and use of animals were considered in the present investigation.

### Test organism

**Fig 1:**
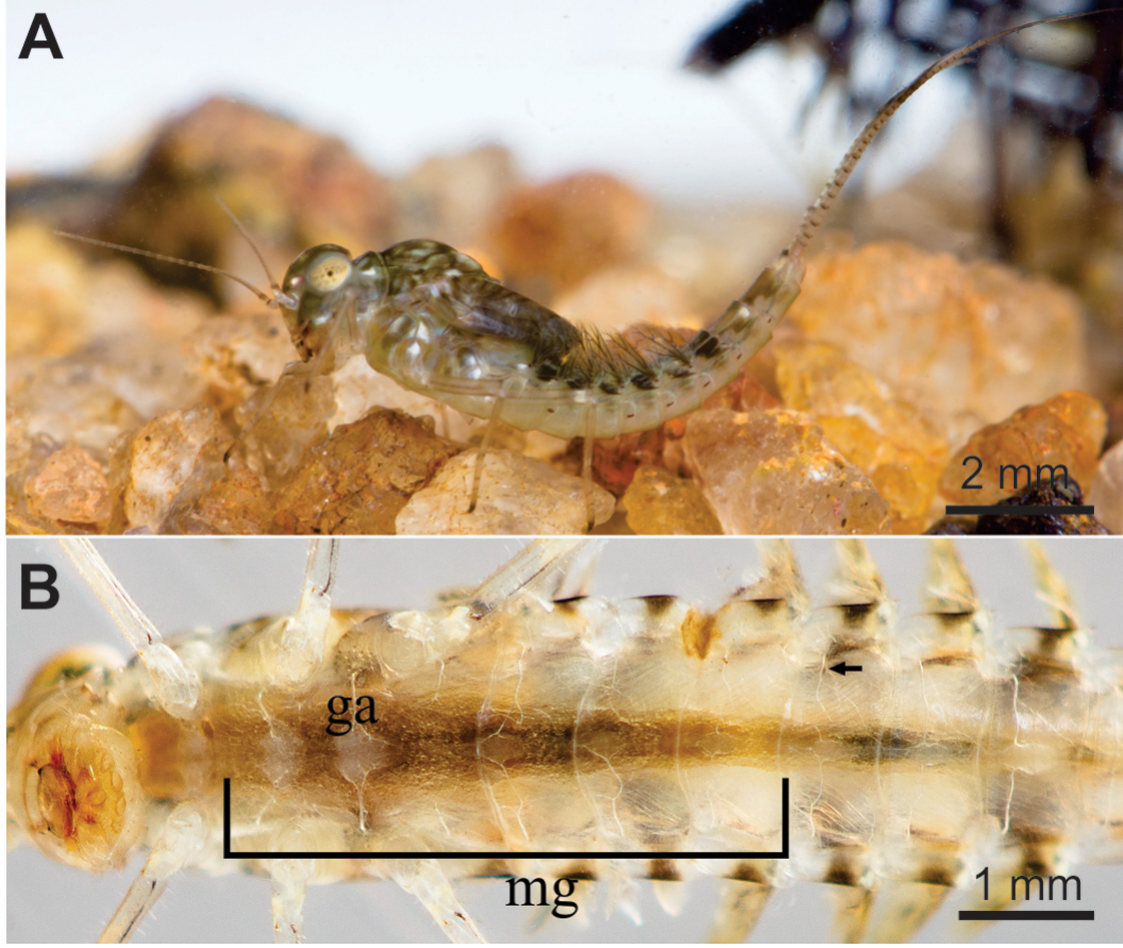
Nymph of *C*. *radiatus*. (**A**). Lateral view (**B**) Ventral view. **ga**: ganglion, **mg**: midgut; **arrow**: trachea.

Nymphs of *C*. *radiatus* (Fig 1) of 7–8 mm size (body length excluding the terminal filaments) were collected with D-net in pesticides-free artificial lakes at the fish-farming station of the Federal University of Viçosa (Viçosa, Minas Gerais State, Brazil). These nymphs were transferred to a laboratory and maintained at 25 ± 2°C, 70 ± 5% relative humidity and 12 h photophase for 24 h before use in the experiments.

### Concentration-mortality bioassays

Groups of *C*. *radiatus* nymphs were exposed to a commercial formulation of deltamethrin (Decis 25EC®, Bayer CropScience Ltda., São Paulo, Brazil) at concentrations that ranged from 0.25 to 5 μg of a.i/L. These deltamethrin concentrations were selected after preliminary tests with a broad concentration range allowing the selection of lower (the highest deltamethrin concentration unable to kill *C*. *radiatus*) and upper (the smallest deltamethrin concentration able to kill 100% of *C*. *radiatus*) mortality responses. In the control treatment, only dechlorinated tap water was used. The exposure time was 24 h and individuals were considered to be dead when no movement of their appendages (legs, antennae, and terminal filaments) or gills was observed after repeated gentle mechanical stimulation with a pipette tip. Thus, the experimental unit consisted of groups of 10 nymphs that were submitted to 0.3 L of solution confined in 0.5 L glass vials (Laborquimi Vidrolabor, São Paulo, Brazil). Five replicates were used for each insecticide concentration.

### Histology

To detect sublethal effects of deltamethrin exposure, groups of 10 *C*. *radiatus* nymphs were exposed to the lower deltamethrin concentration (0.25 μg of a.i/L) and individuals who survived the exposure time for 1, 12 and 24 h were randomly selected to cytomorphological analysis. The control treatment (i.e., without insecticide application) consisted of exposure to dechlorinated tap water. The nymph size and insecticide exposure procedures were similar to the procedures described above. After exposure to deltamethrin, five nymphs of *C*. *radiatus* for each exposure time (1, 12 and 24 h) were dissected (Fig 1B) in the presence of insect saline solution (0.13 M NaCl; 0.01 M; Na_2_HPO_4_ 0.02 M; KH_2_PO_4_; pH 7.2) and their midguts, brains and thoracic ganglia were transferred to Zamboni fixative solution [35] for 2 h. The samples were dehydrated in a graded ethanol series and embedded in JB4 Historesin (Electron Microscopy Sciences, Hatfied, PA, USA). Slices 2 μm thick were stained with hematoxylin and eosin and analyzed with a light microscope (Olympus BX53, Olympus Deutschland, Hamburg, Germany). Some midgut sections were submitted to the P.A.S histochemical tests to for detection of polysaccharides and neutral glucoconjugate [36]. Another set of midgut sections of the nymphs that were 24 h exposed to insecticide were submitted to the Feulgen reaction to evidence DNA [37].

### Apoptosis

#### Immunofluorescence

The midguts, brains and thoracic ganglia from five nymphs that were exposed to deltamethrin (0.25 μg of a.i/L) for 1, 12 and 24 h were dissected in 0.1 M sodium phosphate buffer (PBS) and transferred to Zamboni fixative solution for 15 min, followed by washing with PBS containing 1% Triton X-100 (PBST) and incubation with 1.5% bovine serum albumin in PBST for 15 min. Then, the samples were incubated with anti-cleaved-caspase 3 antibody (Trevigen, Gaithersburg, MD, USA) at 1:500 in PBST for three days. After washing with PBST, the samples were incubated with anti-rabbit IgG FITC-conjugated antibody at 1:500 in PBST for two days in the dark, washed with PBST and the nucleus was stained with iodide propidium (5 mg/mL) for 5 min. The pieces were mounted with 50% sucrose and examined under a laser scanning confocal microscope (LSM510 META, Zeiss, Thornwood, NY, USA).

#### DNA damage

The total DNA was extracted from the midgut, brain and thoracic ganglia from three *C*. *radiatus* nymphs for each insecticide exposure time. The organs were dissected in 0.1 M PBS, homogenized in liquid nitrogen and incubated in the DNA extraction buffer (100 mM Tris–HCl; 25 mM EDTA; 100 mM NaCl; 1% SDS; pH 8.0). The DNA extracts were incubated in 6 μL of proteinase K for 1 h at 60°C for protein degradation. Then, the samples were incubated in ice for 5 min. A phenol/chloroform/ethanol solution (25:24:1) was added, and the samples were centrifuged at 12.000 x *g* for 10 min. The supernatant was collected and mixed with 10 μL of 3 M sodium acetate and 20 μL of ethanol. The DNA was stored at −20°C for 24 h, centrifuged at 12.000 x *g* for 10 min and the pellet washed with 70% ethanol. Finally, the DNA was resuspended in 50 μL of TE buffer (10 mM Tris–HCl; 1 mM EDTA), submitted to electrophoresis in 0.8% agarose gel and stained with GelRed^TM^ (Biotium, Hayward, CA, USA)

### Survival bioassays

Nymphs of *C*. *radiatus* were exposed to four deltamethrin concentrations (0.25, 0.5, 2.5 and 5 μg of a.i/L) determined by the concentration-mortality bioassay or to dechlorinated tap water (control). All the exposure procedures and insect sizes followed the same procedures described above for the concentration-mortality bioassays. The glass vials were covered with organza net to prevent losses of the emerged adults. The number of dead nymphs and emerged adults was recorded every six hours during 4 days (96 h). The dead individuals were removed from the vials as soon as they were registered in order to prevent cannibalism.

### Statistical analysis

Concentration–mortality curves were estimated by probit analyses using the PROC PROBIT procedure [38]. The results of the survival bioassays were subjected to survival analysis using the Kaplan–Meier estimator (Log-rank method) with the SigmaPlot 12.0 software (Systat Software, San Jose, California, USA). The nymphs surviving until the end of the experiment, as well as the emerged adults, were treated as censored data.

## Results

### Concentration-mortality and survival bioassays

The probit model was suitable for the concentration-mortality results with χ2 < 3.0 and *P* > 0.05. Deltamethrin lethal concentrations (LC_20_, LC_50,_ LC_80_ and LC_99_) are shown in Table 1.

**Table 1:** Toxicity of deltamethrin to the mayfly *C*. *radiatus* (*n* = 270, *χ*^2^ = 2.10, *P* = 0.35). Lethal concentration (LC) values were estimated based on concentration-mortality bioassays using probit analyses. CI denotes confidence interval. Concentrations are expressed in μg of a.i/L.

Slope ± SE	LC_20_ (95% CI)	LC_50_ (95% CI)	LC_80_ (95% CI)	LC_99_ (95% CI)
2.03 ± 0.28	0.23 (0.15–0.32)	0.60 (0.46–0.78)	1.56 (1.13–2.25)	3.89 (2.43–8.47)

### Histology

#### Midgut

**Fig 2:**
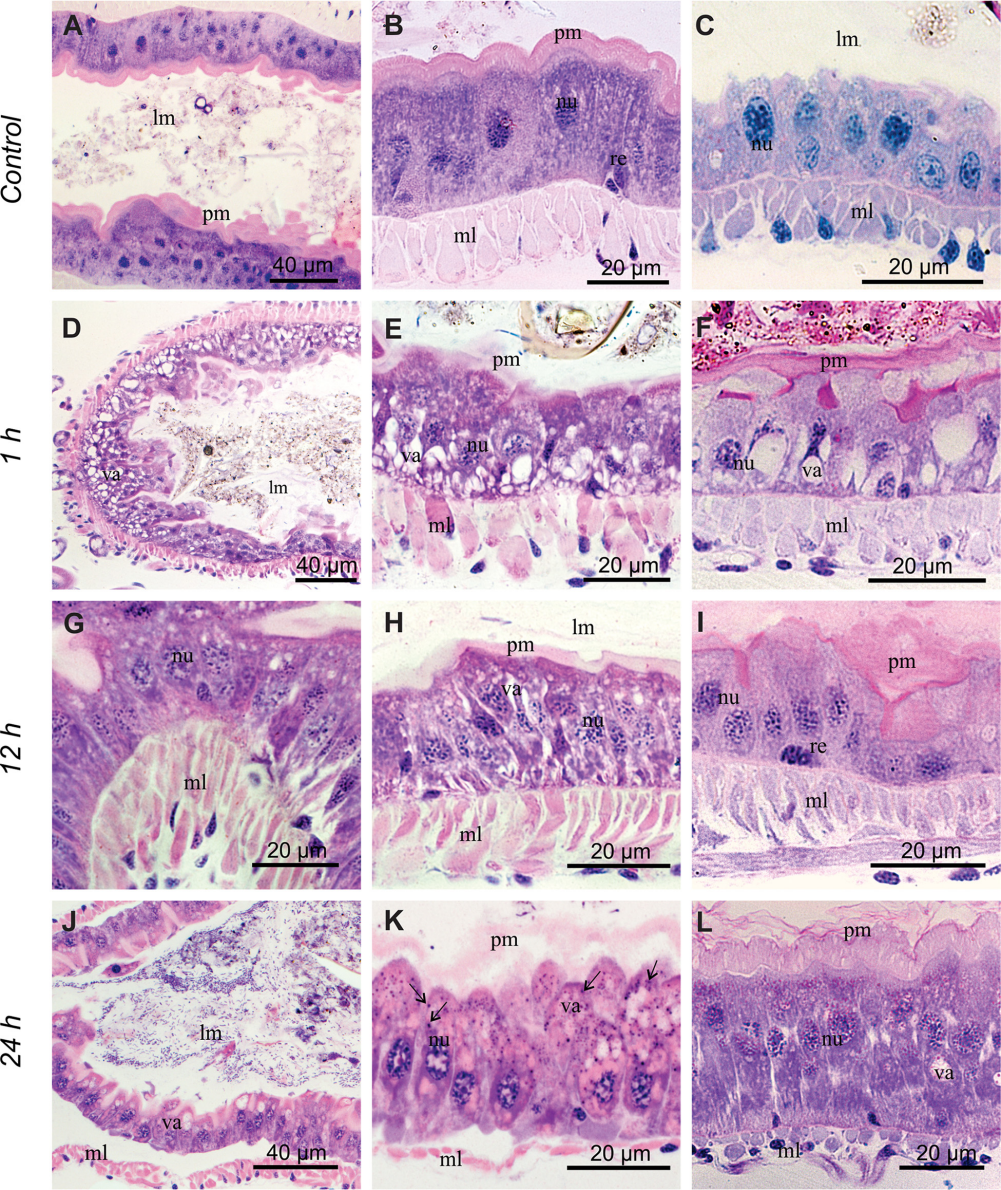
Histological sections of *C*. *radiatus* midgut stained with hematoxylin and eosin. (**A-C**): control treatment with no exposure to insecticide. (**D-F**): 1h exposure to deltamethrin. (**G-I**): 12 h exposure to deltamethrin. (**J-L**): 24 h exposure to deltamethrin. All exposure time were performed for the lowest deltamethrin concentration (0.25 μg of a.i/L) used in this study. **lm**: midgut lumen, **pm**: peritrophic membrane, **nu**: nuclei, **ml**: muscle layer, **va**: vacuolation, **arrows**: basophilic granules.

The midgut of *C*. *radiatus* nymphs not exposed to deltamethrin showed a single-layered epithelium of columnar digestive cells and some scattered nests of regenerative cells (Fig 2A–2C). The digestive cells showed spherical nuclei with some cloths of condensed chromatin (Fig 2A–2C). The apical region of the digestive cells had a well-developed brush border near the peritrophic membrane (Fig 2B) lining the gut content. A well-developed circular muscle layer was found externally to the midgut epithelium (Fig 2B and 2C).

The cytomorphological changes in the midgut were affected by deltamethrin exposure time. The nymphs that survived 1 h of deltamethrin exposure showed columnar digestive cells with higher vacuolization of the basal cytoplasm, brush border and peritrophic membrane weakly acidophilus (Fig 2D–2F). Furthermore, the columnar digestive cell nucleus showed a decrease in the amount of condensed chromatin (Fig 2F). These cytomorphological changes persisted in the survivors of 12 h exposure to deltamethrin and vacuoles with strongly acidophilus content were observed (Fig 2G–2I).

After 24 h of deltamethrin exposure, the cytoplasm of the digestive cells showed decreased vacuolation, the presence of acidophilus vacuoles distributed throughout the cytoplasm and basophilic granules at the apical portion (Fig 2J–2L). The digestive cells re-organized into a columnar epithelium; their nucleus showed a higher amount of condensed chromatin and the cell apex was close to the peritrophic membrane (Fig 2L), whereas the muscle layer was separated from the epithelium (Fig 2K). The peritrophic membrane, brush border and some cytoplasmic granules of digestive cells of *C*. *radiatus* were P.A.S. positive (Fig 2C, 2F, 2I and 2L). Nymphs exposed to deltamethrin for 1 h and 12 h showed a decrease in the amount of P.A.S. positive cytoplasmic granules (Fig 2F and 2I), whereas in those nymphs exposed to deltamethrin for 24 h had P.A.S. positive microgranules in the apical region of the digestive cells (Fig 2L). Some basophilic granules were found in the cytoplasm of digestive cells in 24 h deltamethrin-exposed nymphs (Fig 2K), but these granules were Feulgen negative indicating that they were not nucleus fragments (S1 Fig).

#### Brain and thoracic ganglia

**Fig 3:**
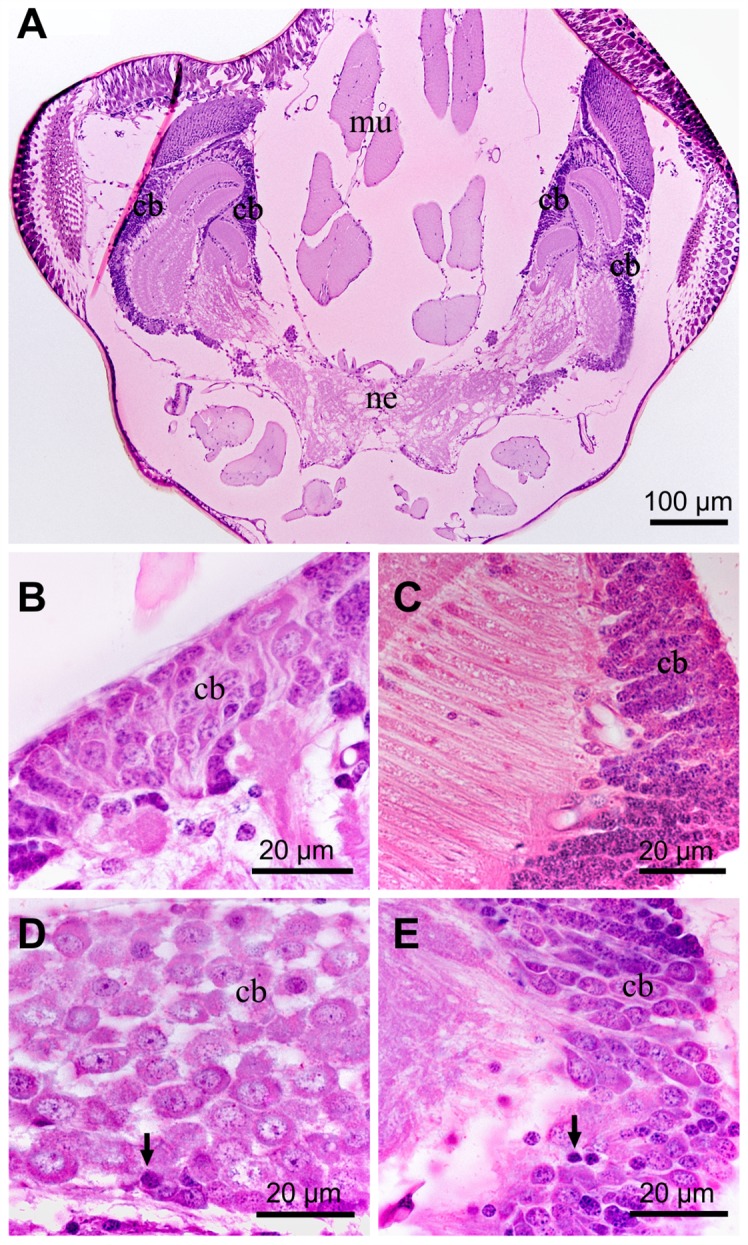
Histological sections of *C*. *radiatus* brain stained with hematoxylin and eosin. (**A**), Whole brain section. (**B**), Control treatment with no exposure to insecticide. (**C**), 1 h exposure to deltamethrin. (**D**), 12 h exposure to deltamethrin. (**E**), 24 h exposure to deltamethrin. All exposure times were performed for the lowest deltamethrin concentration used in this study (0.25 μg of a.i/L). **ne**: neuropile; **cb**: cell body of neuron; **arrow**: pyknotic nucleus; **mu**: muscles.

**Fig 4:**
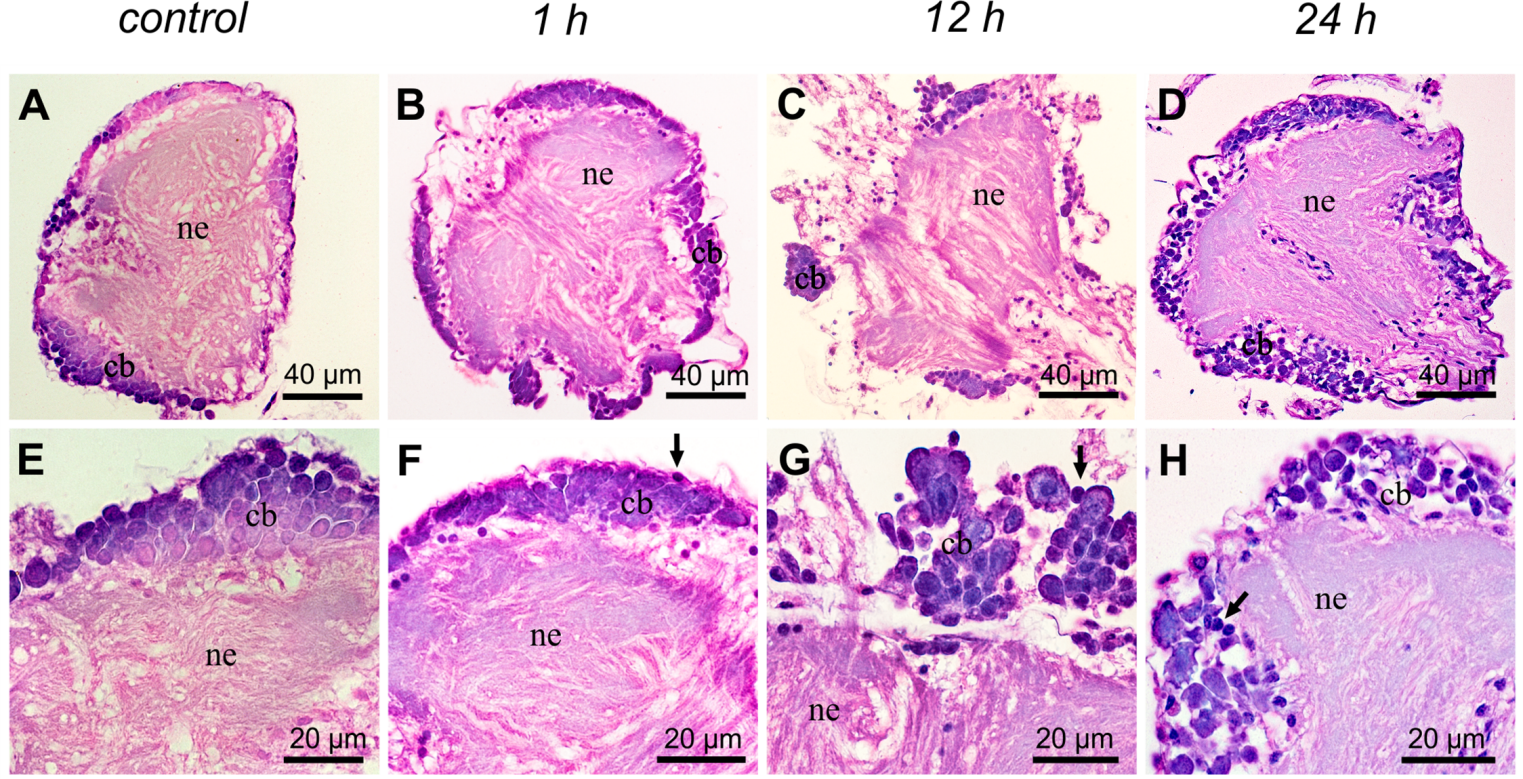
Histological sections of *C*. *radiatus* thoracic ganglia stained with hematoxylin and eosin. (**A** and **E**), Control treatment with no exposure to insecticide. (**B** and **F**), 1h exposure to deltamethsurin. (**C** and **G**), 12 h exposure to deltamethrin. (**D** and **H**), 24 h exposure to deltamethrin. All exposure time were performed for the lowest deltamethrin concentration used in this study (0.25 μg of a.i/L). **ne**: neuropile; **cb**: cell body of neuron; **arrow**: pyknotic nucleus

The brain of *C*. *radiatus* consisted of well-defined regions, showing neuronal cell bodies in the periphery and condensed neuropiles in the central region of the brain (Fig 3A). The protocerebrum, deuterocerebrum and tritocerebrum were evident and the presence of several pyknotic nuclei was the only difference found in the brains of *C*. *radiatus* exposed to deltamethrin (Fig 3A–3E). Significant morphological changes were not found in the thoracic ganglia of unexposed and 1 h deltamethrin-exposed *C*. *radiatus* nymphs (Fig 4A, 4B, 4E and 4F). However, vacuolation in the neuronal cell bodies was observed after 12 h of deltamethrin exposure, which increase after 24 h of deltamethrin exposure (Fig 4C, 4D, 4G and 4H).

### Apoptosis

#### Immunofluorescence and DNA fragmentation

**Fig 5:**
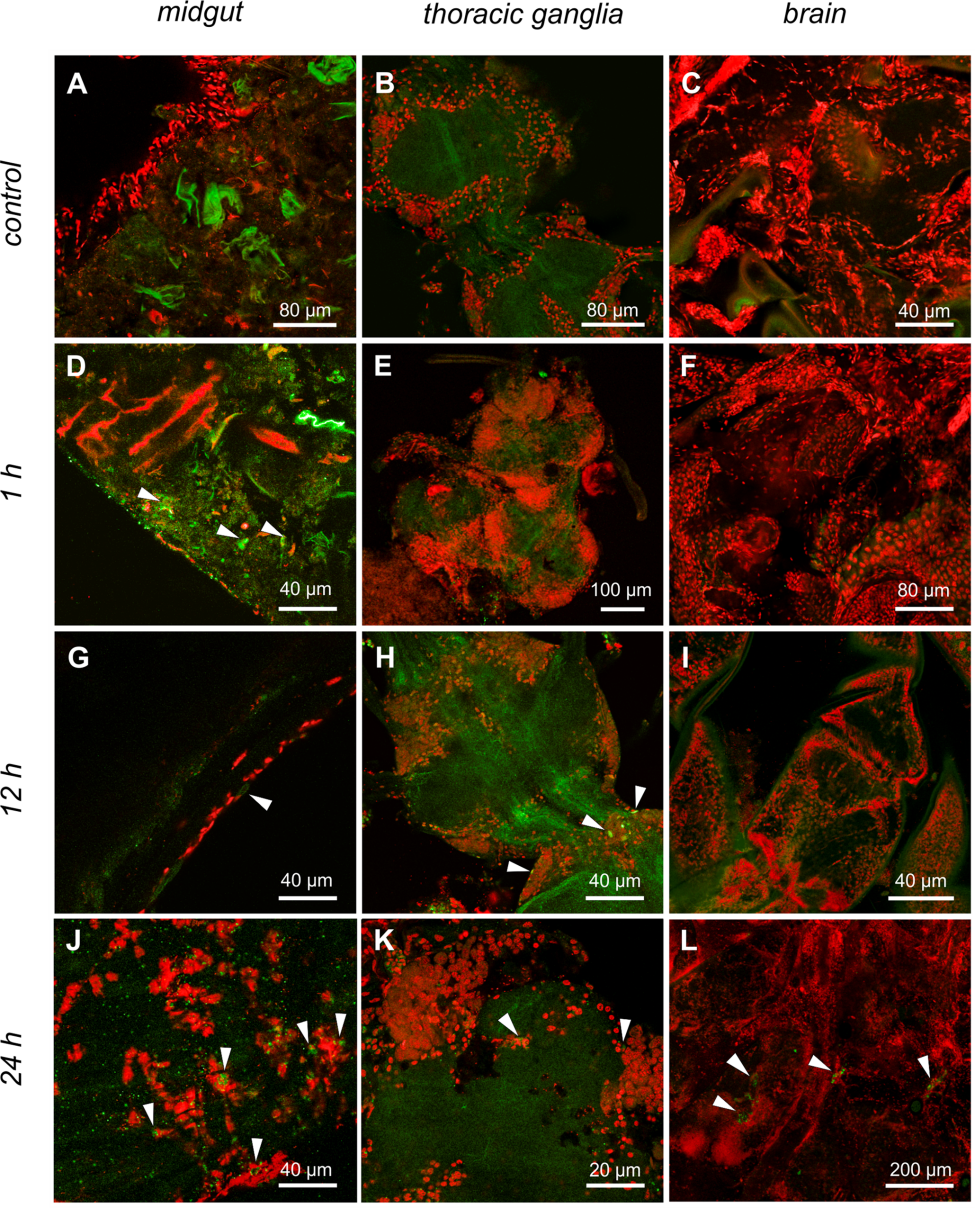
Immunofluorescence of *C*. *radiatus* tissues using anti-cleaved-caspase-3 antibody (green). (**A**, **D, G** and **J**), midgut control, 1, 12, 24 h treatments correspondingly. (**B, E, H** and **K**), thoracic ganglia control, 1, 12, 24 h treatments correspondingly. (**C, F, I** and **L**), brain control, 1, 12, 24 h treatments correspondingly. Arrowheads indicate caspase-3 activity.

In the midgut of unexposed and 1 h deltamethrin-exposed *C*. *radiatus* nymphs, a few cleaved caspase-3 positive digestive cells were randomly distributed (Fig 5A and 5B). Increases in the number of cleaved caspase-3 positive cells were found in only a few regions of the midgut of *C*. *radiatus* nymphs that were 12 h or 24 h deltamethrin-exposed (Fig 5G and 5J)

In the thoracic ganglia of the nymphs that were deltamethrin-exposed for 12 and 24 h, few regions showed nerve cells with a higher cleaved caspase-3 positive reaction (Fig 5H and 5K). In the brain, no cleaved caspase-3 neurons were found for deltamethrin unexposed nymphs, whereas deltamethrin-exposed nymphs showed some cleaved caspase-3 positive neurons, but this was independent of the duration of deltamethrin exposure (Fig 5C, 5F, 5I and 5L). DNA fragmentation was completely absent in the analysis of DNA integrity from the midgut, thoracic ganglia and brain of nymphs exposed to all time treatments, (S2 Fig).

### Survival Analysis

**Fig 6:**
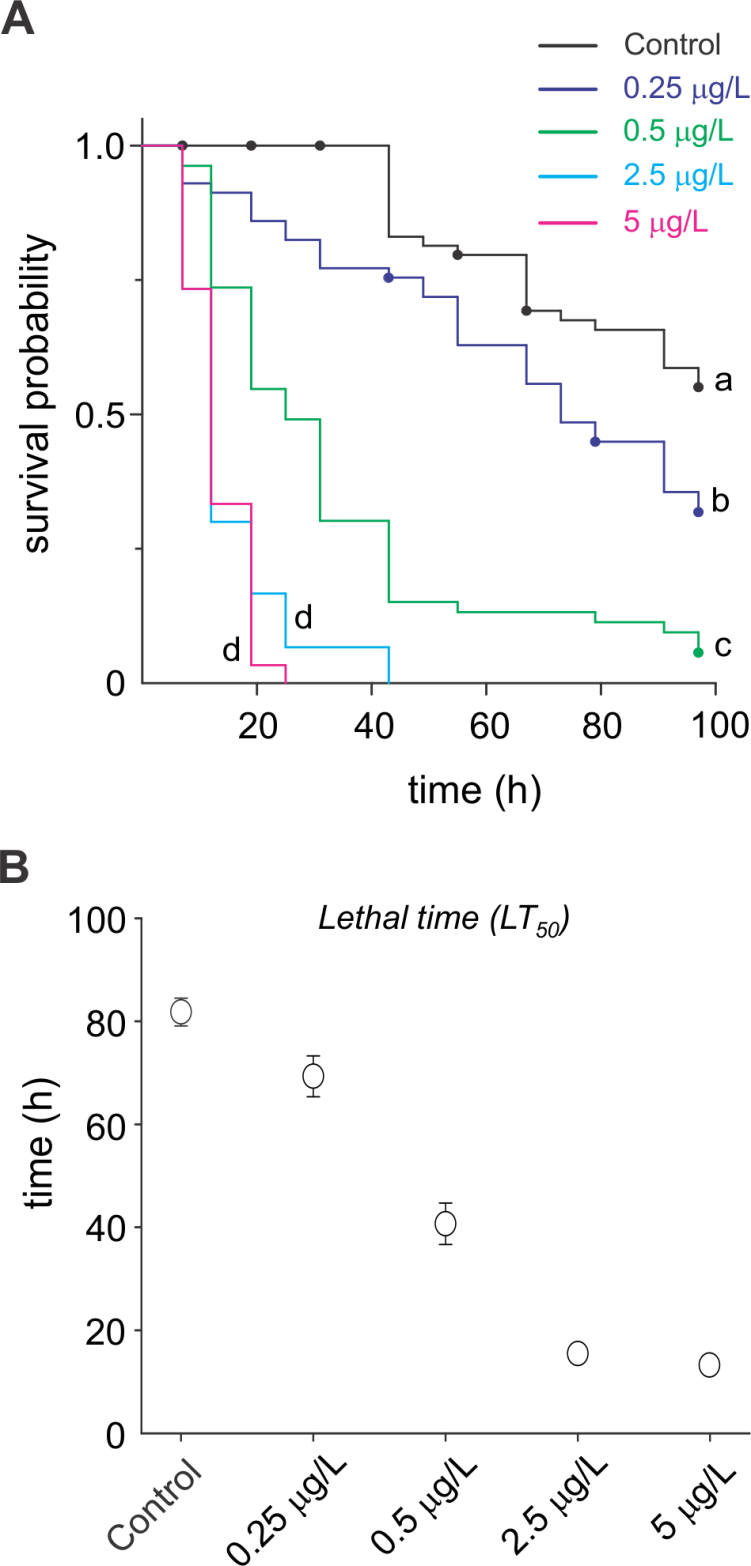
Deltamethrin-mediated changes in the survival abilities of *C*. *radiatus*. (**A**) Survival curves of *C*. *radiatus* nymphs subjected to up to 96 h deltamethrin exposure. Only the survival curves of the highest concentrations (2.5 and 5 μg of a.i/L) were not significantly different by Holm-Sidak’s test (*P* > 0.05). Points represent the censored data (nymphs surviving until the end of the experiment, as well as the emerged adults). (**B**) Mean lifetime of *C*. *radiatus* nymphs under 96 h deltamethrin exposure. Dispersion expressed as SE.

The survival analysis of the data from *C*. *radiatus* nymphs exposed to deltamethrin residues indicated significant differences among the insecticide concentrations (Log-rank test, χ2 = 204.7, df = 4, *P* < 0.001). After four days of exposure, survival was above 60% for nymphs that had not been exposed to deltamethrin, decreasing to approximately 30% at the lowest deltamethrin concentration (0.25 μg of a.i/L) and dropping to less than 6% at the deltamethrin concentration of 0.5 μg of a.i/L (Fig 6A). Deltamethrin concentration higher than 0.5 μg of a.i/L resulted in 100% mortality of nymphs for exposure times <48 h (Fig 6B).

## Discussion

The immature instars of benthic mayflies are among the most sensitive arthropods to pyrethroid insecticides [27,29]. Here, we assessed deltamethrin toxicity to *C*. *radiatus* nymphs (including the effects on their survival abilities) and evaluated the deltamethrin-mediated changes in the digestive and central nervous system of these insects. The nymphs of *C*. *radiatus* were highly susceptible to deltamethrin (LC_50_ = 0.60 [0.46–0.78] μg/L), reducing their survival abilities when exposed to deltamethrin concentration as low as 0.25 μg/L. Short-term (up to 24 h and at 0.25 μg/L) exposure to deltamethrin did not induce cell death in the midgut and central nervous system but affected their cytomorphology, suggesting potential disturbances in the physiological processes (e.g., metabolism or electrical signal transmission) of *C*. *radiatus* that might lead to survival deficits.

The cytomorphological alterations in the insect midgut caused by insecticides have been reported in different insect species [24,26,39–42]. Here, the degeneration of digestive cells (e.g., vacuolation, decreased acidophilia in the brush border and decreased amount of condensed chromatin in the cell nucleus) in the midgut of *C*. *radiatus* nymphs exposed for 1 h and 12 h to deltamethrin seems to be mitigated in the nymphs exposed for 24 h, which suggests that midgut cells undergo some detoxification processes, thereby reducing the deltamethrin effects. The occurrence of detoxification processes in the insect midgut is not unexpected since the primary functions of the midgut include digestive enzyme production and nutrient absorption of digestion products [42–45]. In this sense, larvae of the mosquito *Culex quinquefasciatus* showed higher vacuolization in the midgut cells after exposure to insecticides, including deltamethrin [24].

It is worth to note that *C*. *radiatus* might have an efficient midgut epithelium, because this layer was structured with digestive and regenerative cells, while other ephemeropterans only showed digestive cells [46]. The presence of some cytoplasm basophilic granules in the midgut cells of 24 h deltamethrin-exposed nymphs suggest a potential bioaccumulation of deltamethrin and its byproducts, as suggested elsewhere for other toxicants [47,48]. One might suggest that the basophilic granules are nucleus fragments commonly found in cells that undergo apoptosis, but we showed by the Feulgen reaction that these granules are not of nuclear origin.

In addition to the afore-mentioned changes, cytoplasm vacuolization in the midgut of *C*. *radiatus* might represent the initial processes of deltamethrin-mediated changes, which ultimately may result in autophagy [49–51], apocrine secretion and/or apoptosis [52–54]. However, the measurements of cleaved caspase-3 positive cells, which are extensively used as an indicator of apoptosis in animal cells [54–56], only slightly increased in the midgut of deltamethrin-exposed nymphs. These cleaved caspase-3 cell increases might indicate an early stage of the apoptosis process in the assessed organs, but it cannot be ruled out that this phenomenon may be part of the normal cell renewal in these organs [56,57]. This latter hypothesis is supported by the absence of DNA fragmentation in the organs of *C*. *radiatus* exposed to deltamethrin.

The brain and thoracic ganglia of *C*. *radiatus* nymphs show the usual morphology described for other ephemeropterans [58] with the brain presenting proto- deuto- and tritocerebrum regions and the thoracic ganglia, likely having a cortex region formed by the neuron cell bodies and the central region (neuropile) formed by the axon and dendrites. Although major pyrethroid actions occur on the axonal nerve impulse transmission [17–19], here we found only slight changes in the cytomorphology of the nerve cells in the brain and in the thoracic ganglia. On contrary, the synganglion of the *Rhipicephalus sanguineus* (Acari: Ixodidae) females showed strong structural and enzymatic changes after exposure to the pyrethroid permethrin [59]. Such differential susceptibility might reflect differences between the protection efficacy provided by the perineurium and glial cells in insects and mites, once this cell layers prevent or decrease the interaction of insecticide molecules with the neural environment [59–61].

The differences of severity and type of cytomorphological changes among the organs studied may result from the deltamethrin toxicological characteristics. Deltamethrin also causes secondary effects that contribute to its toxicity [19,62,63]. For instance, osmotic imbalances in the digestive tract may contribute to deltamethrin secondary actions, since the digestive tract is one of the main routes of absorption and accumulation in heterotrophic aquatic organisms [64,65]. Exposure to sublethal deltamethrin concentrations results in cytomorphological changes that may reduce the animal’s ability to convert ingested food into the nutrients needed for their development and/or reproduction [65,66].

The survival of *C*. *radiatus* nymphs was reduced under longer exposure (up to 96 h) to a deltamethrin concentration that did not induce cell death up to 24 h of exposure. When exposed to sublethal concentrations of a pesticide, the basal metabolism of insects could increase in response to the physiological stress imposed by the pesticide [67], which might not affect the ability to survive a single insecticide pulse but will certainly increase the chance to make the insect less healthy [68]. For instance, the sublethal exposure of arthropods to deltamethrin have been showed to impair their gas exchange system, decreasing the oxygen uptake and dysregulation of the breathing activities [69]. Thus, our findings show that the cytomorphological alterations found in the deltamethrin (0.25 μg a.i./L; 24 h exposure period)-exposed mayflies represent a clear indication of sublethal effects on the *C*. *radiatus* survival abilities, which might reduce the densities of *C*. *radiatus* populations and, consequently, disturbing the lentic macroinvertebrate assemblages where this insecticide is used (e.g. near agricultural and urban areas).

## Supporting Information

S1 Data Raw data used in the Probit analysis. (PDF)

S2 Data Raw data used in the survival analysis. (PDF)

S1 Fig Histological sections of *C*. *radiatus* midgut after 24 h exposure to deltamethrin stained with Feulgen technique. **lm:** midgut lumen, **nu**: nuclei, **ml**: muscle layer.
(PDF)

S2 Fig DNA integrity of the midgut (MG), thoracic ganglia (Gan) and brain (Brn) of *C*. *radiatus* for the unexposed (cont.) and the 1, 12, and 24 h exposure treatments. The first column corresponds to the standard (S).
(PDF)
