# Supplementary material for: Deltamethrin-Mediated Toxicity and Cytomorphological Changes in the Midgut and Nervous System of the Mayfly Callibaetis radiatus
Source: PLoS One. 2016 Mar 31;11(3):e0152383. doi: 10.1371/journal.pone.0152383 (PMC4816402; doi:10.1371/journal.pone.0152383)
Supplement: S1 Data — (PDF) [file pone.0152383.s001.pdf]

Supporting Informations_S1 : Raw data used in the Probit Analysis

concentrations	Dead	Total
0	0	11
0	0	13
0	0	12
0	0	10
0	0	10
0	0	10
250	1	11
250	1	15
250	3	11
250	4	10
250	1	10
500	2	10
500	7	12
500	5	11
500	9	10
500	4	10
2500	7	10
2500	10	10
2500	9	10
5000	9	10
5000	10	10
5000	10	10
