# Supplementary material for: Deltamethrin-Mediated Toxicity and Cytomorphological Changes in the Midgut and Nervous System of the Mayfly Callibaetis radiatus
Source: PLoS One. 2016 Mar 31;11(3):e0152383. doi: 10.1371/journal.pone.0152383 (PMC4816402; doi:10.1371/journal.pone.0152383)
Supplement: S2 Data — (PDF) [file pone.0152383.s002.pdf]

Supporting Informations_S2 : Raw data used in the Survival Analysis

0	1	alive
0	7	censored
0	12	alive
0	19	censored
0	19	censored
0	25	alive
0	31	censored
0	31	censored
0	43	dead
0	43	dead
0	43	dead
0	43	dead
0	43	dead
0	43	dead
0	43	dead
0	43	dead
0	43	dead
0	43	dead
0	49	dead
0	55	dead
0	55	censored
0	67	dead
0	67	dead
0	67	dead
0	67	dead
0	67	dead
0	67	dead
0	67	censored
0	73	dead
0	79	dead
0	91	dead
0	91	dead
0	91	dead
0	91	dead
0	97	dead
0	97	dead
0	97	censored
0	97	censored
0	97	censored
0	97	censored
0	97	alive
0	97	censored
0	97	censored
0	97	censored
0	97	censored
0	97	censored
0	97	censored
0	97	censored

0	97	censored
0	97	censored
0	97	censored
0	97	censored
0	97	censored
0	97	censored
0	97	censored
0	97	censored
0	97	censored
0	97	censored
0	97	censored
0	97	censored
0	97	alive
0	97	alive
0	97	censored
0	97	censored
0	97	censored
0	97	censored
0	97	censored
0	97	censored
0	97	censored
0	97	censored
250	1	alive
250	7	dead
250	7	dead
250	7	dead
250	7	dead
250	12	dead
250	19	dead
250	19	dead
250	19	dead
250	25	dead
250	25	dead
250	31	dead
250	31	dead
250	31	dead
250	43	censored
250	43	dead
250	49	dead
250	49	dead
250	55	dead
250	55	dead
250	55	dead
250	55	dead
250	55	dead
250	67	dead
250	67	dead
250	67	dead
250	67	dead
250	73	dead

250	73	dead
250	73	dead
250	73	dead
250	79	dead
250	79	dead
250	79	censored
250	91	dead
250	91	dead
250	91	dead
250	91	dead
250	91	dead
250	97	dead
250	97	dead
250	97	censored
250	97	alive
250	97	censored
250	97	censored
250	97	censored
250	97	alive
250	97	censored
250	97	censored
250	97	censored
250	97	censored
250	97	censored
250	97	alive
250	97	censored
250	97	censored
250	97	censored
250	97	alive
250	97	alive
250	97	alive
250	97	censored
250	97	censored
250	97	censored
250	97	censored
250	97	censored
500	1	alive
500	7	dead
500	7	dead
500	12	dead
500	12	dead
500	12	dead
500	12	dead
500	12	dead
500	12	dead
500	12	dead
500	12	dead
500	12	dead
500	12	dead
500	12	dead

500	12	dead
500	19	dead
500	19	dead
500	19	dead
500	19	dead
500	19	dead
500	19	dead
500	19	dead
500	19	dead
500	19	dead
500	19	dead
500	25	dead
500	25	dead
500	25	dead
500	31	dead
500	31	dead
500	31	dead
500	31	dead
500	31	dead
500	31	dead
500	31	dead
500	31	dead
500	31	dead
500	31	dead
500	43	dead
500	43	dead
500	43	dead
500	43	dead
500	43	dead
500	43	dead
500	43	dead
500	43	dead
500	49	alive
500	55	dead
500	67	alive
500	73	alive
500	79	dead
500	91	dead
500	97	dead
500	97	dead
500	97	alive
500	97	alive
500	97	alive
500	97	alive
500	97	censored
500	97	censored
500	97	alive
500	97	alive
500	97	censored
2500	1	alive

2500	7	dead
2500	7	dead
2500	7	dead
2500	7	dead
2500	7	dead
2500	7	dead
2500	7	dead
2500	7	dead
2500	12	dead
2500	12	dead
2500	12	dead
2500	12	dead
2500	12	dead
2500	12	dead
2500	12	dead
2500	12	dead
2500	12	dead
2500	12	dead
2500	12	dead
2500	12	dead
2500	12	dead
2500	19	dead
2500	19	dead
2500	19	dead
2500	19	dead
2500	25	dead
2500	25	dead
2500	25	dead
2500	31	alive
2500	43	dead
2500	43	dead
2500	49	--
2500	55	--
2500	67	--
2500	73	--
2500	79	--
2500	91	--
2500	97	--
5000	1	alive
5000	7	dead
5000	7	dead
5000	7	dead
5000	7	dead
5000	7	dead
5000	7	dead
5000	7	dead
5000	7	dead
5000	12	dead
5000	12	dead
5000	12	dead

5000	12	dead
5000	12	dead
5000	12	dead
5000	12	dead
5000	12	dead
5000	12	dead
5000	12	dead
5000	12	dead
5000	12	dead
5000	19	dead
5000	19	dead
5000	19	dead
5000	19	dead
5000	19	dead
5000	19	dead
5000	19	dead
5000	19	dead
5000	19	dead
5000	25	dead
5000	31	--
5000	43	--
5000	49	--
5000	55	--
5000	67	--
5000	73	--
5000	79	--
5000	91	--
5000	97	--
