# Supplementary figures and images for: Deltamethrin-Mediated Toxicity and Cytomorphological Changes in the Midgut and Nervous System of the Mayfly Callibaetis radiatus
Source: PLoS One. 2016 Mar 31;11(3):e0152383. doi: 10.1371/journal.pone.0152383 (PMC4816402; doi:10.1371/journal.pone.0152383)

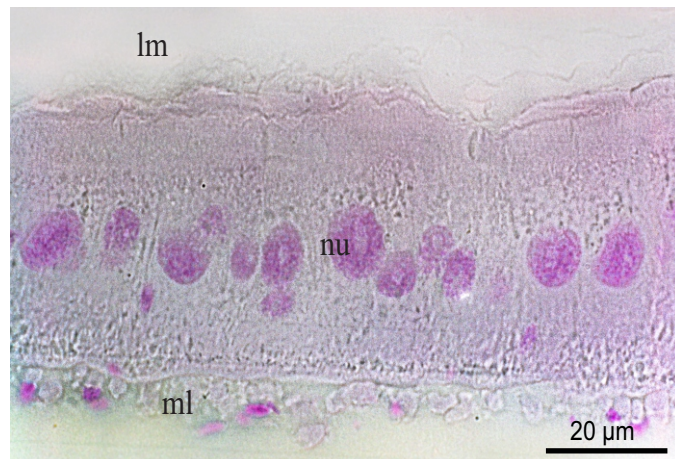

Supplementary Figure 1

Supplement: S1 Fig — lm: midgut lumen, nu: nuclei, ml: muscle layer. (PDF) [file pone.0152383.s003.pdf]

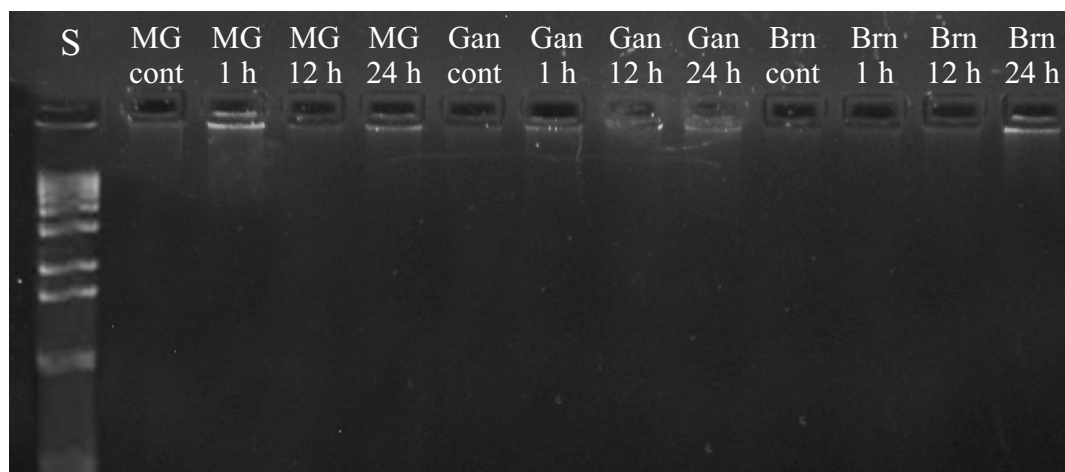

Supplementary Figure 2

Supplement: S2 Fig — The first column corresponds to the standard (S). (PDF) [file pone.0152383.s004.pdf]
